# Supplementary material for: Revisiting the contribution of different factors in determining the changes in potential evapotranspiration over China
Source: PLoS One. 2024 Apr 16;19(4):e0299468. doi: 10.1371/journal.pone.0299468 (PMC11020870; doi:10.1371/journal.pone.0299468)
Supplement: Attachment — Submitted filename: Response to Reviewer.docx [file pone.0299468.s003.docx]

Response to Reviewer1

1. Line 17-18: Authors are kindly requested to provide detailed information regarding the range of values depicting both the increase and decrease in Potential Evapotranspiration (PET) throughout the summer and winter seasons.

Response: Thank you for your suggestions. Based on your opinion, we have updated the manuscript to reflect the range of climatology and trend values in Potential Evapotranspiration (PET) throughout the summer and winter seasons in Line 27-28 and 30-32 in manuscript.

“PET reaches its peak during summer which values from 145 to 640 mm, while it is lowest during winter from 21 to 244 mm.”

“The reduction is most significant during the summer and the range of trend is from -2.04 to 1.48 mm/day, while the increase becomes more pronounced in the winter which trend is from -0.34 to 0.53 mm/day.”

2. Line 22: what are the little factors or mention the common factors

Response: Thank you for your suggestions. In spring and autumn, RH and U have little difference in contribution from other factors. But sun hours are varying different from regions, the contribution value is largest in the northwest and smallest in the northeast. We have revised in manuscript from line 32-34 in manuscript.

“In spring and autumn, RH and U have little difference in contribution from other factors. But tsun is varies different from regions, the contribution value is largest in the northwest and smallest in the northeast”

3. Line 30 what are the five components of the climate system

Response: We apologize for not clarified this issue clearly. The five components of climate system are the atmosphere, the hydrosphere, the cryosphere, the lithosphere and the biosphere. We have revised in manuscript from line 40-41 in in manuscript.

“The interactions and interdependence among the five components of climate system, includes the atmosphere, the hydrosphere, the cryosphere, the lithosphere and the biosphere, are of great importance.”

4. Line 44: Ref?

Response: Thank you for your suggestions. This content can be found in the reference 9, and we have revised in manuscript in line 55 in manuscript.

5. Line 51: cite the recent studies conducted on the spatial and temporal on the PET in China to back up the points

Response: Thank you for your suggestions. We have added recent relevant references (15-17) about spatial and temporal distribution of PET in China to the manuscript in line 63 in manuscript.

6. Line 80: what is the name of the novelty methods approaches employed to the study.

Response: Thank you for your suggestions. We used multiple regression coefficients to calculate the relative contribution of different meteorological factors to trend changes. The revised text can be found in Line 93-94 in manuscript.

“Secondly, this study examines the relative contributions of various meteorological factors to changes in PET at a national scale, utilizing multiple regression coefficients to calculate the relative contribution of different meteorological factors.”

7. Line 85: how to understand?

Response: we have changed "builds upon" to "complements". Please see Line 99 in manuscript.

“In summary, this study complements previous research by investigating PET trends at a daily scale across China's six subregions and examining the relative contributions of meteorological factors to PET changes.”

8. Figure 1, The map show should indicate the spatial elevation or ecological variation of each zone and should be in color

Response: Thanks for your suggestion. We have replaced Figure 1 with a figure containing elevation information.

Response to Reviewer2

This research study was conducted on the topic “Revisiting the contribution of different factors in determining the changes in Potential evapotranspiration over China” which seems to be very interesting. It considers daily scale PET in China across different zones and its contributing factors. It also gives ideas about trends in historical periods across the region. However, the authors used previous methods used by other people. Therefore, it is required to give more justification for certain results produced in a research paper. Furthermore, grammatical proofreading should be done by some experts to increase the drafting quality of the paper. I recommend minor to major comments for authors to improve the quality of research. I have given the comments in the Adobe Reader file. However, the authors are suggested to look into the following studies conducted on regional PET calculations and their related themes. These can give more ideas to authors to further clarify their results and interpretation of results more effectively.
1. <https://doi.org/10.1007/s00382-023-06831-6>
2. Spatiotemporal Analysis of Groundwater Resources Sustainability in South Asia and China using GLDAS Datasets." (2022).

Response: We are very grateful for the Reviewer’s detailed comments which are valuable and helpful for improving our manuscript. We have incorporated all the suggestions and comments in the revised manuscript.

1. Line 57: Please add: "condition" after arid.

Response: Thank you for your suggestions. we have revised in manuscript in line 70 in manuscript.

“The contribution of evapotranspiration to climate change is particularly important in northern China, where the climate is dominant by the arid condition.”

2. Line 80: This is not innovative method. This method already applied. But you can say " this knowledge gape is completed using these methods or something like this.

Response: Thanks for your suggestions. We used multiple regression coefficients to calculate the relative contribution of different meteorological factors to trend changes. We have revised it in line 92-95 in manuscript.

“Secondly, this study examines the relative contributions of various meteorological factors to changes in PET at a national scale, utilizing multiple regression coefficients to calculate the relative contribution of different meteorological factors. This approach allows for a comprehensive assessment of the influence of different meteorological factors on PET changes.”

3. Figure 1: Study area map should be revised showing some elevations would be more attracting.

Response: Thanks for your suggestion. We have replaced Figure 1 with a figure containing elevation information.

4. Line 141: Multiple linear regression method should be described more. Because this is important part of analysis.

Response: Thank you for your suggestion. We have made a brief introduction of multiple linear regression method in the revised manuscript in line 156-167 in manuscript.

5. Line 153: Before explaining figures. Please explain something about the reason why that figure or analysis was carried out. It would be better to explain the reason of analysis and then the explanation about figures.

Response: Thanks for your patient guidance. Different regions in China have different climate characteristics. Understanding the distribution of PET climatology in these different regions can help us understand its changes in the region better. We have revised the manuscript in line 179-180 in manuscript.

“Different regions in China have different climate characteristics. Understanding the distribution of PET climatology in these different regions can help us understand its changes in the region better.”

6. Line 158: correct it to “more”

Response: Thanks for your patient guidance. We have revised it.

7. Line 166: Can you please explain why NC receiving highest PET and in spring PET in SC became lower.

Response: Thank you for your questions. We think the highest PET in Northwest China in summer is mainly due to the combination effect of longer sunshine hours, higher average temperature and lower relative humidity than other regions.

Relative humidity is higher in southwest China in summer than spring, in my opinion this may be one of the reasons why PET is smaller in summer than spring.

We have revised in line 194-195 and line 197-198 in manuscript.

“Because of the combination effect of longer sunshine hours, higher average temperature and lower relative humidity than other regions, Northwestern China received the highest PET values, with majority of regions reporting PET values exceeding 360mm.”

“Unlike in spring, the PET values in southwest China are comparatively lower than those in regions at the same latitude, this may due to higher relative humidity than other regions.”

8. Line 173: Please try to explain why this is higher in southeaster coastal region

Response: Thank you for your suggestions. We think this is possibly influenced by the ocean at low latitude, the southern coastline exhibits higher PET. we have added in Line 203 in manuscript.

“Notably, the PET in the southeastern coastal region is higher compared to areas at the same latitude, this is may partly due to the influence of ocean at low latitude.”

9. Line 193: Please rewrite sentence and correct grammatical mistakes.

Response: Thanks for your patient guidance. We have rewritten this sentence in line 224-226 in manuscript.

“In general, the trends remain relatively consistent across the four seasons. The eastern regions predominantly witness a decreasing trend, whereas the western regions predominantly show an increasing trend.”
